# Fish Skin-Derived Acellular Matrix with pH-Modulating Nanogels for Wound Healing

**DOI:** 10.34172/apb.46166

**Published:** 2026-05-10

**Authors:** Hanieh Mahdiani, Faegheh Yazdani, Haleh Bakhshandeh, Hossein Aminianfar, Nazanin Samiei, Sholeh Maslehat, Amir Amanzadeh, Mahsa Khoramipour, Shahram Azari, Mohammad Ali Shokrgozar, Rassoul Dinarvand

**Affiliations:** ^1^Department of Pharmaceutical Nanotechnology, Faculty of Pharmacy, Tehran University of Medical Sciences, Tehran, Iran; ^2^Nanotechnology Research Centre, Faculty of Pharmacy, Tehran University of Medical Sciences, Tehran, Iran; ^3^Nanobiotechnology Department, New Technologies Research Group, Pasteur Institute of Iran, Tehran, Iran; ^4^Department of Pathobiology, Faculty of Veterinary Medicine, University of Tehran, Tehran, Iran; ^5^National Cell Bank of Iran, Pasteur Institute of Iran, Tehran, Iran

**Keywords:** Acellular dermal matrices, Fish skin, Polyelectrolyte complex, Wound healing, pH modulation, Nanogels

## Abstract

**Purpose:**

Fish skin–derived acellular dermal matrices (FS-ADMs) are promising wound dressings due to their low zoonotic risk and minimal chemical processing. This study investigated the combined effects of pH modulation as a chemical intervention and FS-ADM as a physical scaffold on wound healing. Carp skin was selected for its favorable lipid composition and high exudate absorption capacity.

**Methods:**

Hyaluronic acid (HA)–based nanogels composed of HA and L-lysine, with or without tannic acid (TA), were synthesized and characterized using FTIR spectroscopy and light scattering. Full-thickness excisional wounds were created in 30 Wistar rats and evaluated over 21 days for re-epithelialization, collagen organization, neovascularization, inflammation, and appendage regeneration. Animals were divided into five groups: negative control, FS-ADM alone (SC), FS-ADM with HA nanogel (SC+NC), and FS-ADM with two TA-loaded HA nanogel formulations (SC+NP1 and SC+NP2). Data were analyzed using GraphPad Prism 9.0.

**Results:**

The nanogels exhibited sustained acidifying behavior, reducing pH by approximately 1.5 units over seven days in vitro. Degradation studies showed scaffold breakdown of 37% within three days and 60% by day 21, indicating suitable biodegradation kinetics. Medicated FS-ADMs demonstrated excellent biocompatibility, maintaining 96% L929 fibroblast viability. The SC+NC group exhibited the most effective healing (9.67±0.58), achieving complete epithelialization, organized collagen deposition, and sebaceous gland regeneration by day 21. The SC+NP2 group also showed substantial healing, including hair follicle regeneration. HA nanogel without TA outperformed TA-loaded systems, suggesting a dominant role of HA in enhancing tissue repair.

**Conclusion:**

Combining FS-ADM with pH-modulating nanogels significantly accelerates wound healing through sustained acidification and represents a promising strategy for chronic wounds such as diabetic ulcers.

## Introduction

 The skin is the body’s largest organ, serving as a protective barrier against mechanical damages and infections, while also regulating fluid balance and body temperature.^1^ Maintaining healthy skin is vital for overall physiological stability, making effective wound care essential.^2^ Wound healing is a complex process involving three key stages: inflammation, proliferation, and maturation.^3^ Research has shown that pH play a crucial role in this process. As healing progresses, the wound environment typically shifts from alkaline to acidic. This acidic shift can enhance the effectiveness of wound dressings, and hyaluronic acid has been found to offer multiple benefits in this context.^4^

 The pH of wound exudate is a valuable indicator of both infection and healing progress.^5-9^ Chronic wounds often exhibit a highly alkaline environment, with a pH level of up to 10, conditions that favor the growth of pathogenic bacteria. Therefore,monitoring wound pH can help identify bacterial colonization early and guide timely treatment.^6,7^ Various pH sensors, such as colorimetric and electrochemical types, have been developed and incorporated into bandages for clinical use. Interestingly, the type of dressing applied influences the pH beneath it. Non-permeable dressings tend to create a more acidic environment compared to permeable ones. In vitro studies have shown that acidic wound secretions from occlusive synthetic dressings can suppress bacterial growth while simultaneously promoting fibroblast activity, which is crucial for tissue regeneration.^9^

 Tissue engineering scaffolds are specialized materials designed to perform complex physical and biological functions both inside the body (in vivo) and in laboratory settings (in vitro). Typically made from polymer-based biomaterials, these scaffolds offer structural support for cells to attach and grow, aiding in tissue development.^10^ Nanogels are nanometer-sized hydrogel particles, composed of three-dimensional networks of cross-linked polymer chains. They hold a large amount of water and swell like traditional hydrogels, making them particularly suitable for skin drug delivery.^11^ Hyaluronic acid (HA) is a preferred component due to its viscoelastic nature, excellent biocompatibility, and non-immunogenic properties.^12^ HA is a linear anionic polysaccharide made of a repeating disaccharide units: β-D-glucuronic acid and N-acetyl β-D-glucosamine.^13^ It supports wound healing by promoting nutrient transport, removing waste through interaction with CD44 receptors, and maintaining hydration. Its strong water-binding capacity also enhances skin moisture elasticity.^14^ L-lysine is another important molecule, known for its role in tissue repair and protein function.^15^ Studies have shown that L-lysine positively influences cell growth and improves the mechanical strength of polymeric scaffolds.^16^ Cellular enzymes involved in tissue repair and regeneration, such as Poly (ADP-ribose) polymerases (PARPs), are highly sensitive to environmental and intracellular changes. For instance, “Effects of a Cancer-Associated Mutation and Multiple Serine Phosphorylation on Poly (ADP-Ribose) Polymerase 2” demonstrated how post-translational modifications regulate PARP2 activity, influencing DNA repair and cellular recovery processes. Such molecular-level regulation underscores how biochemical environments, including pH fluctuations, can impact cellular repair efficiency during wound healing.^17^

 Acellular dermal matrix (ADM) is widely used as a wound dressing and skin substitute because of its excellent biocompatibility with human tissue and its ability to promote regeneration.^18^ Traditionally sourced from land animals, ADM can sometimes provoke immune responses.^19^ In contrast, fish skin-derived ADM (FS-ADM) and fish collagen are gaining popularity due to their low immunogenicity and reduced risk of transmitting prion and viral infections.^20^

 Polyelectrolyte complexes (PECs) are charged macromolecules that dissolve in polar solvents due to electrostatic interactions between water molecules and their charged monomers.^21^ These complexes form through the association of particles with opposite charges.^22^ In recent years, PECs have gained considerable attention for o their potential in biomedical applications, particularly in drug delivery. They enhance drug properties such as stability and solubility by encapsulating drugs at the molecular level. PECs also offer advantages like biocompatibility and the absence of harmful crosslinking agents.^23^ Incorporating pH-regulating components into PEC-based nanostructures allows controlled acid release, helping maintain a stable acidic environment on wound surface. This is crucial for infection control, which plays a key role in promoting wound healing.^24^

 In this study, a scaffold was developed using carp fish skin, chosen for its high protein and fat content that supports tissue regeneration. The scaffold was infused with a nanoparticle complex that combining L-lysine and HA, with tannic acid added to the nanogel to regulate pH levels. The nanogel was thoroughly characterized, and its acid-releasing ability was tested in vitro to evaluate its effectiveness in lowering pH. To ensure the scaffold’s purity, FS-ADM was examined for the removal of nucleoporins and then immersed in the nanogel solution to enable physical bonding. The study concluded with in vivo testing on rats, focusing on pH changes, wound contraction, and tissue-level healing in full-thickness excisional wounds on the dorsal skin.

## Material and Methods

###  Materials

 The skins of carp fish were purchased from Shahrvand Chain Store Co. (Tehran, Iran). Female Wistar rats (9–10 weeks old, weighing 180–200 g) were purchased from the Pasteur Institute of Iran (Tehran, Iran). All animal procedures were conducted in general accordance with the guidelines of the Institutional Animal Care and Use Committee. L929 cells (NCBI C161) were obtained from the cell bank of the Pasteur Institute of Iran. All culture media and reagents were purchased from Gibco Life Technologies Co. (USA), unless specified. Tannic Acid (TA) was purchased from Molecular GmbH (Shanghai, China), and Hyaluronic Acid (HA) and Lysine (Lys) were also supplied by Sigma-Aldrich (UK). All materials and solvents used were of analytical grade. This study was performed in the Cell Bank and Nanotechnology Departments of the Pasteur Institute of Iran.

###  Preparation and Characterization of Nanogels

 The nanogel was synthesized based on a previously published study conducted by our research by the dripping method as follows: 1 ml of TA aqueous solution (8 mg/ml) was added to 2 ml of Lys aqueous solution (27 mg/ml), and then the prepared solution was added dropwise with 30 sec. interval to 2 ml of HA aqueous solution (0.35 mg/ml). All steps were performed on a stirrer at room temperature. After stirring for 30 minutes using the first method of sedimentation, 0.2925 g of NaCl powder was added to the formulation on the stirrer, and stirring continued for an additional 5 minutes. Then, it was centrifuged at 12,000 rpm for 30 minutes at 24 °C (ThermoScientific^TM^ SL16R, ThermoFisher Scientific, Waltham, MA, USA) to collect sedimented nanogels. Supernatants were discarded, and pellets were resuspended in 0.9% sodium chloride LVP. In the second method, the formulation was lyophilized.^25^

####  Fourier-Transform Infrared Spectroscopy

 Fourier transform infrared spectroscopy (FTIR) was recorded with a Frontier^TM^ FT-IR spectrometer (Perkin-Elmer, USA) using the KBr method with a spectral resolution of 1 cm⁻¹ in the range of 400–4000 cm⁻¹.

####  Light Scattering

 Particle size (detection angle of 90°, room temperature) and zeta potential (ZP) measurements were performed using a Zetasizer Nano S90 (Malvern Instruments, Malvern, UK). The analyses were performed in triplicate for each batch.

####  Nanogels Morphology

 Morphological analysis of selected nanogels was performed using a high-resolution scanning electron microscope (JEM-1011, JEOL USA Inc., Peabody, MA, USA). Samples were dried prior to observation. SEM was used to examine the morphology of nanogels. SEM micrographs of vitrified aqueous colloids revealed that the majority of particles had a spherical form and were nanoscale in size. However, some of the objects were made up of two or three particles that were most likely fused during the manufacturing process.

####  Acid Release Studies

 First, the nanogels obtained from three reactions were precipitated in a centrifuge at 12,000 rpm for 15 minutes at 24 °C. This sediment contained 2.4 micrograms of tannic acid in the nanogel formulation. To simulate the release conditions similar to those of a wound surface, the minimum amount of release fluid was used. Then, this sediment was dissolved in a minimum volume of 3 ml of water for injection (pH 6.82) in a microtube obtained from the drugstore. It was then placed in a sonicator for 30 seconds, and the initial pH of the nanogel solution was recorded. The prepared nanogel solution was then placed in a shaker incubator at 37 °C with shaking at 50 rpm. At time intervals of 1, 2, 3, 4, 5, and 24 hours, samples were taken to measure pH changes. After each sampling, each sample was returned to the microtube. To ensure that the plateau between 4 and 24 hours did not indicate equilibrium, the supernatant was removed by centrifugation at 12,000 rpm for 15 minutes at 24 °C and replaced with fresh water for injection. Sampling and measurement of pH continued at intervals of 25, 26, 28, 72, and 144 hours.

###  Fabrication of FS_ADM and Physicochemical Properties

####  ADM Preparation Steps

 The descaled fish skin was separated from the fillet, retaining as much epidermal fat as possible. The descaled skin was washed twice with sterile phosphate buffer. The fish skin was placed in 1 M NaCl for 8 hours, followed by incubation in 2% deoxycholic acid containing 0.02% sodium azide and 500 ppm streptomycin. The solution was poured off, and the fish skin was placed in Hanks’ Balanced Salt Solution (HBSS) for 10 minutes, after which the solution was aspirated off. Then it was treated with a decellularizing solution containing 0.5% SDS on a shaker. The decellularizing solution was aspirated. The fish skin was washed with Dulbecco’s phosphate-buffered saline (DPBS). The fish skin was incubated for 18 hours in a digestion solution that consisted of 1 M Tris-HCl and trypsin at 0.05 µg/mL. For cryoprotection, the fish skin was immersed in a pre-freezing solution containing dextran, sucrose, and 1 mM EDTA in Hank’s Balanced Salt Solution. Then the fish skin was washed with the pre-freezing solution and placed on the shaker. All of the above steps were performed at room temperature. The fish skin was freeze-dried^26^ and sterilized using 25 kg of gamma radiation.

####  Hematoxylin-Eosin Staining

 Carp skin and Acellular Dermal Matrix (ADM) were fixed with 4% (w/v) paraformaldehyde, dehydrated, and embedded in paraffin. Five micrometer (μm) slices were cut, stained with Hematoxylin-Eosin (H&E) staining solution, and observed under a light microscope (Olympus, Japan).

####  Scanning Electron Microscope

 The structures of ADM were observed using a scanning electron microscope (SEM). The ADM was cut into small pieces measuring 1 mm × 1 mm and fixed onto the grid with conductive adhesive. After the surface was sprayed with gold, the microstructure of the ADM was observed using SEM (AIS2100, SERON TECHNOLOGY).

####  Water Absorption Capacity

 The lyophilized specimens were placed in a microtube and then weighed to an accuracy of 0.01 g on a balance. The specimens were then placed into 2 mL of distilled water at 24 °C. During the first day of immersion, the specimens were removed at intervals of 1, 2, 3, 6, and 24 hours, blotted dry on filter paper to remove excess water, weighed, and returned to the water. The samples were weighed hourly until the uptake slowed and there was no significant change in weight. The uptake of water was recorded until equilibrium was attained.^27^

####  Degradation Test

 The lyophilized samples measuring 0.5 cm × 2 cm (n = 4) were cut, accurately weighed (W0), and incubated in 2 mL of Simulated Body Fluid (SBF) at pH 7.37 and 37 °C for 24, 48, and 72 hours. The degraded samples were then rinsed with distilled water, dried using a concentrator for a fixed period, and weighed (Wt) to an accuracy of 0.00001 g on a balance. At each time interval, we used the following equation to calculate the percentage of degradation rate: The percentage of degradation rate (%) = (W0 - Wt) / W0 × 100%.^28^

####  Cytotoxicity and Proliferation Rate

 In this research, L929 cells (NCBI C161) were obtained from the cell bank of the Pasteur Institute of Iran. After refreezing, the cells were added to a flask containing Dulbecco’s Modified Eagle’s Medium (DMEM) culture medium supplemented with 10% Fetal Bovine Serum (FBS), and then the flask was placed in an incubator at 37 °C with 90% humidity and 5% carbon dioxide. It is important to note that the culture medium should be changed every 3 to 4 days.

 The extraction process is based on the ISO 5-10993 standard. For each sterile 3 square centimeter sample, one milliliter of culture medium was added. Then, after 3 days, the environment was removed and added to the cells. A specific amount of culture medium (DMEM) is also used as a control.

 In this study, to investigate the cell proliferation rate, first, 1 x 10^4 cells with 100 microliters of culture medium were added to each well of a 96-well cell culture plate and then incubated for 24 hours at 37 °C so that the cells adhered to the bottom of the plate. After ensuring cell adhesion, as much culture medium as possible was removed from the cells and 90 microliters of the extract were added to each sample. Additionally, 10 microliters of FBS were added to each culture well, and the cells were cultured for another 24 hours. After that, the culture medium was removed, and 100 microliters of 0.5 mg/mL MTT were added to each well before being placed in the incubator for 4 hours. After 4 hours, the solution was removed from the cells, and isopropanol was added to better dissolve the purple crystals of MTT sediment. The plate was then placed on a shaker for 15 minutes. Then, the concentration of the substance dissolved in isopropanol was calculated by reading OD using an ELISA reader (BioTek ELx808, USA) at a wavelength of 570 nm. The well with a higher number of cells shows higher optical density (OD) than wells with fewer cells. Therefore, the relationship between OD and cell concentration can be determined and compared with the control sample according to these equations:

 Toxicity% = (1-mean OD of sample/mean OD of control) *100

 Viability% = 100- toxicity%

###  Preparation of FS-ADM Loaded with HA or TA Nanogels

 To prepare the samples, 1.0 × 1.0 cm sections of the fish skin-derived acellular dermal matrix (FS-ADM) were incubated in each nanogel solution separately, for 1 hour at room temperature to promote physical interaction between the ADM and the nanogel components.

###  In Vivo Animal Study

####  Rat Full-Thickness Skin Defects

 Female Wistar rats (Pasteur Institute, Tehran, Iran; 9–10 weeks old, weighing 180–200 g) were enrolled in this study. They were housed in individual cages at controlled temperature and humidity with a 12-hour light-dark cycle, and had ad libitum access to food and tap water. Animal anesthesia was performed with an intraperitoneal injection of ketamine (100 mg kg−1) from Bremer Pharma GmbH and xylazine 2% purchased from Alfas an. After anesthesia, animals were randomly classified into five groups, and the excisional wound splinting model was created as previously described.^29^ In brief, the hairs on the dorsal surface of the rats were shaved after anesthetizing. A 15-mm Acu-Punch kit (Acuderm Inc., USA) was used to create full-thickness excisional skin wounds on the dorsum of each rat.^29,30^ After tissue-engineered graft transplantation and preparation of the wound healing model, rats were randomly divided into five groups including: group 1: Control SC (n = 6), group2: SC + NC (n = 6), group 3: SC + NP1 (n = 6), group 4: SC + NP2 (n = 6), and group 5: Neg. Control.

####  Measurement of Surface ph

 Scaffold pH was measured after treatment with medicinal or blank solutions and before suturing. Then, at zero time after suturing the ADM, and during 1, 2, 3, 7, and 14 days, the surface of the wound was measured using a ZenTest® PH60F-Z Smart Flat pH Tester Kit.

####  Macroscopic Assessment

 Digital photographs of the wounds were taken at the defined time points. In the rat model, the wound areas were measured using ImageJ software, and the percentage of wound closure at different time points was calculated according to Equation (1).


(1) Wound closure (%)=(A0−At)/A0×100%

In which: A0 = the original wound area at day 0; At = the open wound area at a given time.

####  Histological Assessment

 Tissue samples were collected from the wound area on days 3, 7, 14, and 21 post-wounding. The samples were fixed in 10% neutral buffered formalin, dehydrated through graded alcohol series, embedded in paraffin blocks, and sectioned at 5 μm thickness. Following standard histological procedures, the sections were stained using Hematoxylin and Eosin (H&E) and Masson’s Trichrome methods. The stained tissue sections underwent qualitative (descriptive) assessment of the healing process and semi-quantitative evaluation through scoring healing indices on days 14 and 21. A comprehensive scoring system was used to evaluate six key healing parameters: re-epithelialization, epidermal thickness index, keratinization, granulation tissue quality, scar elevation index, and tissue remodeling.^31^ The total wound healing score was calculated by summing individual parameter scores and finally analyzed statistically.^24^

####  Ethical Considerations

 Animal studies were conducted according to the codes of ethics declared by the Iran National Committee for Ethics in Biomedical Research (IR.TUMS.TIPS.REC.1398.210). According to procedures approved by the Ethics Committee of Tehran University of Medical Sciences, Tehran, Iran, to reduce animal suffering, we aimed to minimize the number of animals used in this research.

###  Statistical Analysis

 All data were analyzed using the GraphPad Prism 9.0 program. One-way analysis of variance (ANOVA) was used, and differences were considered significant at the level of *P* ≤ 0.05.

## Results

###  Nanogel Preparation and Characterization

####  Nanogels Morphology

 The morphology of nanogel was visualized by SEM. SEM micrographs of vitrified aqueous colloids are presented in Figure S1A, B. It was observed that the main population of particles had a rather spherical shape and nanoscale size. However, some of the particles consisted of two or three particles, most likely fused during the synthesis procedure.

####  Fourier-Transform Infrared Spectroscopy

Figure S1C illustrates the FTIR spectra of TA, HA nanogel, and the TA-loaded HA nanogel polyelectrolyte complex. The spectral region 3600 and 3000 cm−1 primarily reflects O-H and N-H stretching vibrations within the nanogel matrix. Characteristic CH2 symmetric and asymmetric stretching bands were detected near 2930 and 2850 cm–1 in the HA nanogel spectrum.^32^ Additional peaks at 2588 cm–1 and 2544 cm–1 were associated with the presence of quaternary ammonium salt. Notably, these spectral features were also present in the TA loaded nanogel, albeit with minor shifts. Distinct absorption bands at 1712 cm−1 and 1612 cm−1 were attributed to C = O stretching of the acetyl group (amide I). A broad, intense band at 3466 cm−1 indicated the O-H stretching vibration of TA. The peaks at 1712 cm−1 and 1612 cm−1 further corresponded to C = O and C = C functional groups of TA.^33,34^ The observed changes in peak intensity and position of in the TA-loaded nanogels likely result from hydrogen bonding interactions within the complex.

####  TA Release Studies

 Within 4 hours, pH decreased by ~1 unit (8.7→7.65) likely due to the release of acidic constituents such as TA and HA from the nanogel matrix. Over the subsequent 24 hours, the system reached a state of equilibrium, resulting in a plateau in the release profile (Figure S1D). Upon replacing the supernatant with f fresh medium, the pH continued to decline gradually, reaching a near-neutral value of 7.04 by 144 hours (Figure S1D). Notably, after 72 hours, the formation of aggregated plaques was observed, possibly caused by alteration in particle surface charge leading to aggregation. The release experiment was conducted in triplicate under identical conditions, and the data were systematically analyzed.

###  Decellularization of Carp Fish Skin

 Decellularization is essential for eliminating residual nucleic acids from ADM, as these molecules act as antigens and may provoke a foreign body response.^35^ To assess the successful removal of nuclei from Carp fish skin, both histological staining and DNA quantification were conducted. Figure 1 displays the H&E staining results. In the native carp skin samples, residual nuclei were clearly visible as blue-stained regions (Figure 1A and 1B). In contrast, no nuclei were detected in the FS-ADM samples (Figure 1C, D). Additionally, the DNA content in FS-ADM was measured at 7.4 ± 0.5 ng per mg of dry tissue, well below the industry-accepted threshold of 50 ng/mg.^35^ These findings confirm the efficiency of the decellularization protocol used for carp skin, which aligns with the method described by Lau CS et al involving the combined use of nuclease and SDS.^35,36^

**Figure 1:**
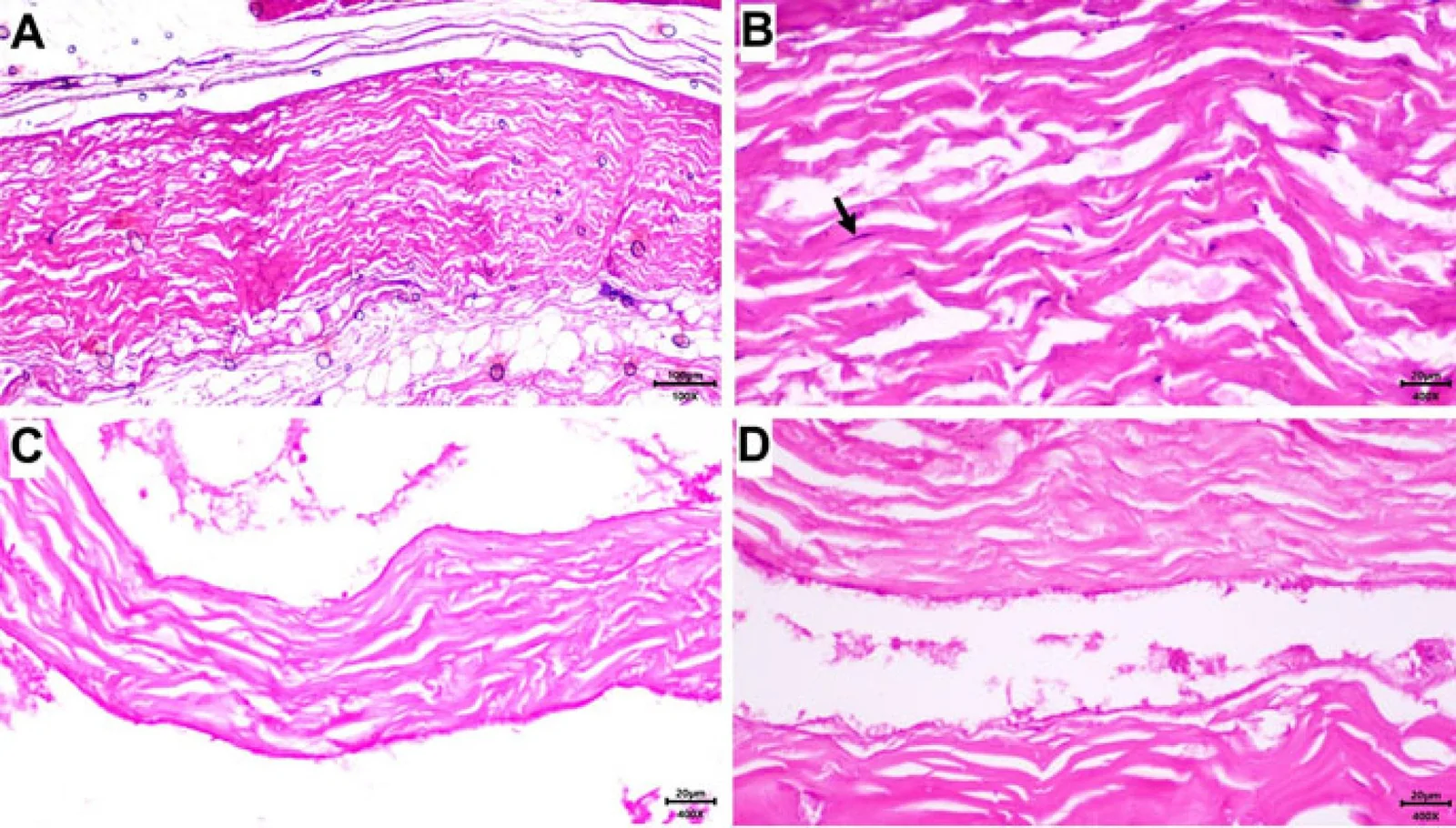
Histological comparison of native carp skin and fish skin-derived acellular dermal matrix (FS-ADM). H&E staining of native carp skin (A: 100X, B: 400X) demonstrates intact tissue architecture with distinguishable epidermis and hypodermis in panel A. In panel B (400X), cellular structures are visible, with fibrocytes (indicated by black arrow) embedded within the collagen fiber network of the dermis. H&E staining of the FS-ADM (C: 100X, D: 400X) confirms successful decellularization, with panel C showing complete removal of epidermal and hypodermal layers, leaving only the dermal matrix. Panel D (400X) demonstrates the absence of cellular components while preserving the native collagen fiber architecture and structural integrity of the extracellular matrix

###  Characterization of Physicochemical Properties of FS-ADM

####  Morphology

Figure 2A-C illustrate the microstructural features and physical attributes of the material, an asymmetric morphology and a multilayered fibrous configuration. Although the FS-ADM sample exhibited some voids, its surface appeared smooth and consistent. The presence of surface indentations and elevations facilitated the infiltration of nanogels into these cavities.

**Figure 2:**
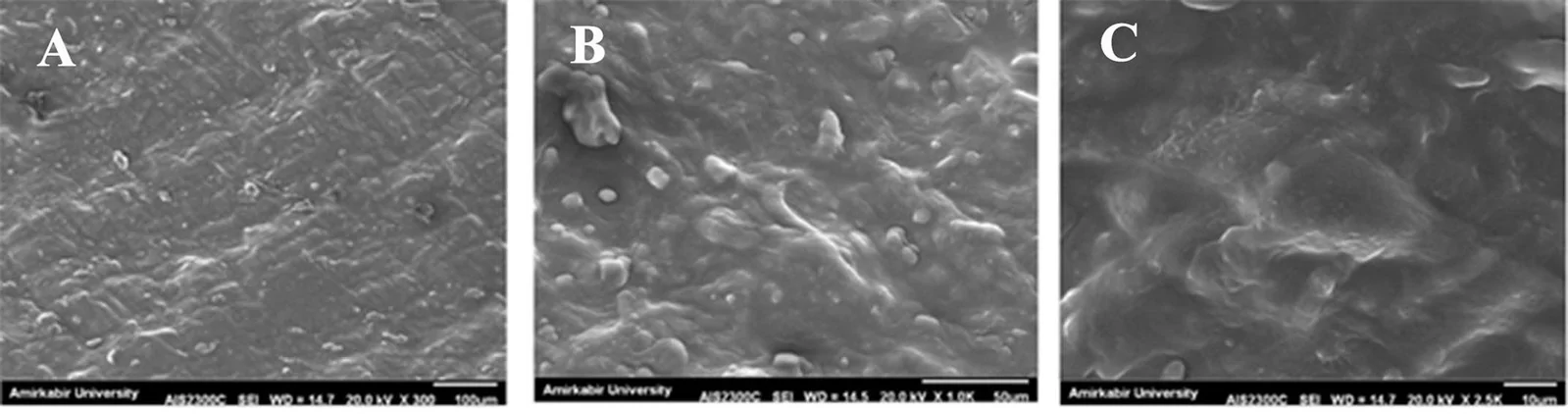
SEM images of Carp fish ADM (A) the scale bar represents 100µm and (B) the scale bar represents 50µm (C) the scale bar represents 1µm

####  Water Absorption Capacity

 The water absorption capacity of the carp fish-derived material was assessed over a period of 24 hours (Figure S2A). Notably, within the first three hours, the material absorbed nearly 100% of its capacity, indicating excellent liquid uptake and retention during this initial phase. This property is particularly beneficial in the early stages of wound healing, where efficient management of exudate is essential for preserving a moist environment conductive to tissue repair and preventing damage to surrounding skin. Furthermore, the material maintained its high absorption rate, approximately 92%, even after 24 hours, suggesting prolonged functionality and reducing the need for frequent dressing replacements, thereby minimizing disturbance at the wound site.

####  Degradation Test

 The degradation behavior of carp-derived FADM in simulated body fluid (SBF) revealed a rapid initial breakdown, with the degradation rate reaching approximately 37% within the first three days (Figure S2B). Following this, the rate of degradation slowed but continued to rise gradually to about 60% over a 21-day period. There was a steady rise to about 60%. Overall, the data reflects a biphasic degradation profile, an initial burst followed by sustained, gradual decline. This pattern is advantageous for some applications, such as wound dressings or r tissue engineering scaffolds, where an early release of bioactive agents is beneficial, followed by prolonged material stability and controlled release over time.

###  Biocompatibility Evaluation

Figure 3 presents the cytotoxicity assessment of the test group against L929 cells. As shown, the test material did not induce any cytotoxic effects when compared to the control group. The SC + NP group showed a mean cell viability of 98.66%, represented by the prominent black bar, indicating minimal impact on cell health. Although the control group, displayed a slightly higher cell viability, the difference is negligible, and both results reflect excellent cell viability these finding confirm that the scaffold and nanogel components in the test formulations are safe and biocompatible

**Figure 3:**
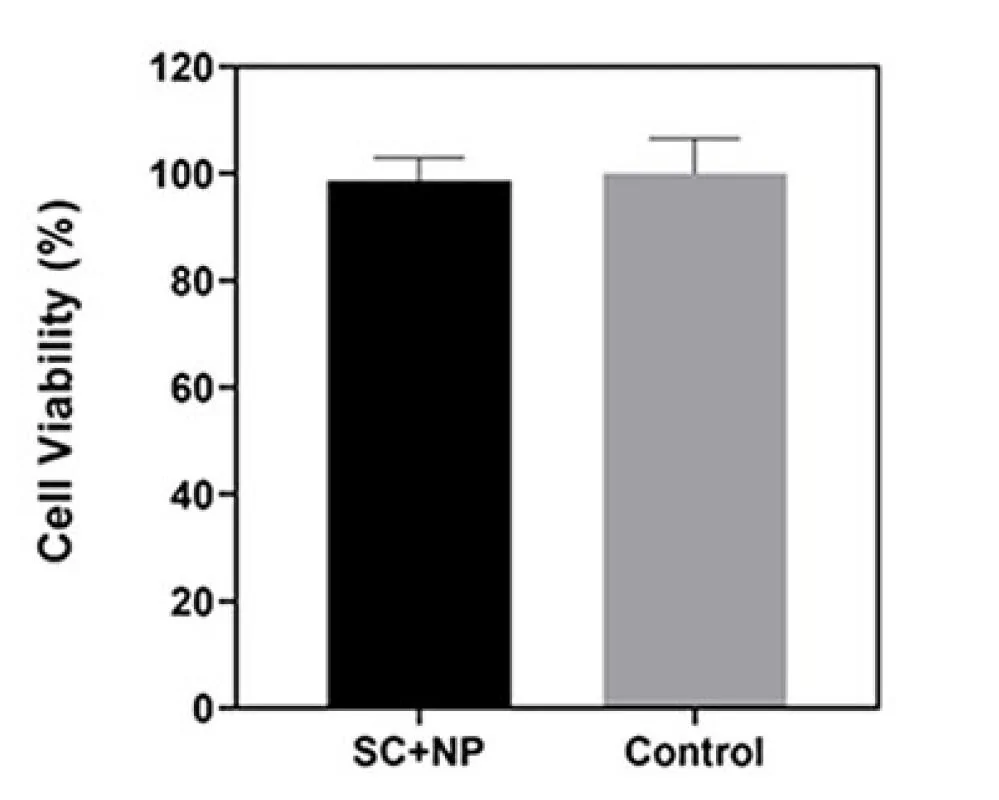
Cell viability of L929 fibroblasts after 24 hours of culture on FS_ADM (control) and FS-ADM loaded with TA nanogel (SC + NP), assessed by the MTT assay. Data are presented as mean ± standard deviation (SD) from three independent experiments. No significant difference was observed between the groups (*P* > 0.05)

###  In Vivo Animal Study

####  The Trend of in Vivo Ph Changes

 Throughout the study period, the control SC group maintained a relatively stable pH, with values ranging from 7.36 to 8.715 (Figure 4A). In contrast, the initial pH levels in the SC + NC, SC + NP1, and SC + NP2 groups were noticeably higher than those in the negative control, likely due to the influence of scaffold and nanogel components on the local pH environment. Over time, a gradual decline in pH was observed in all three experimental groups, which may be attributed to the breakdown or release of these components. Notably the SC + NC group reached its lowest pH value of 6.68 at 72 hours, likely resulting from the rapid release of HA from the nanogel into the surrounding tissue. These findings suggest emerging trends and highlight the promising potential of the tested material.

**Figure 4:**
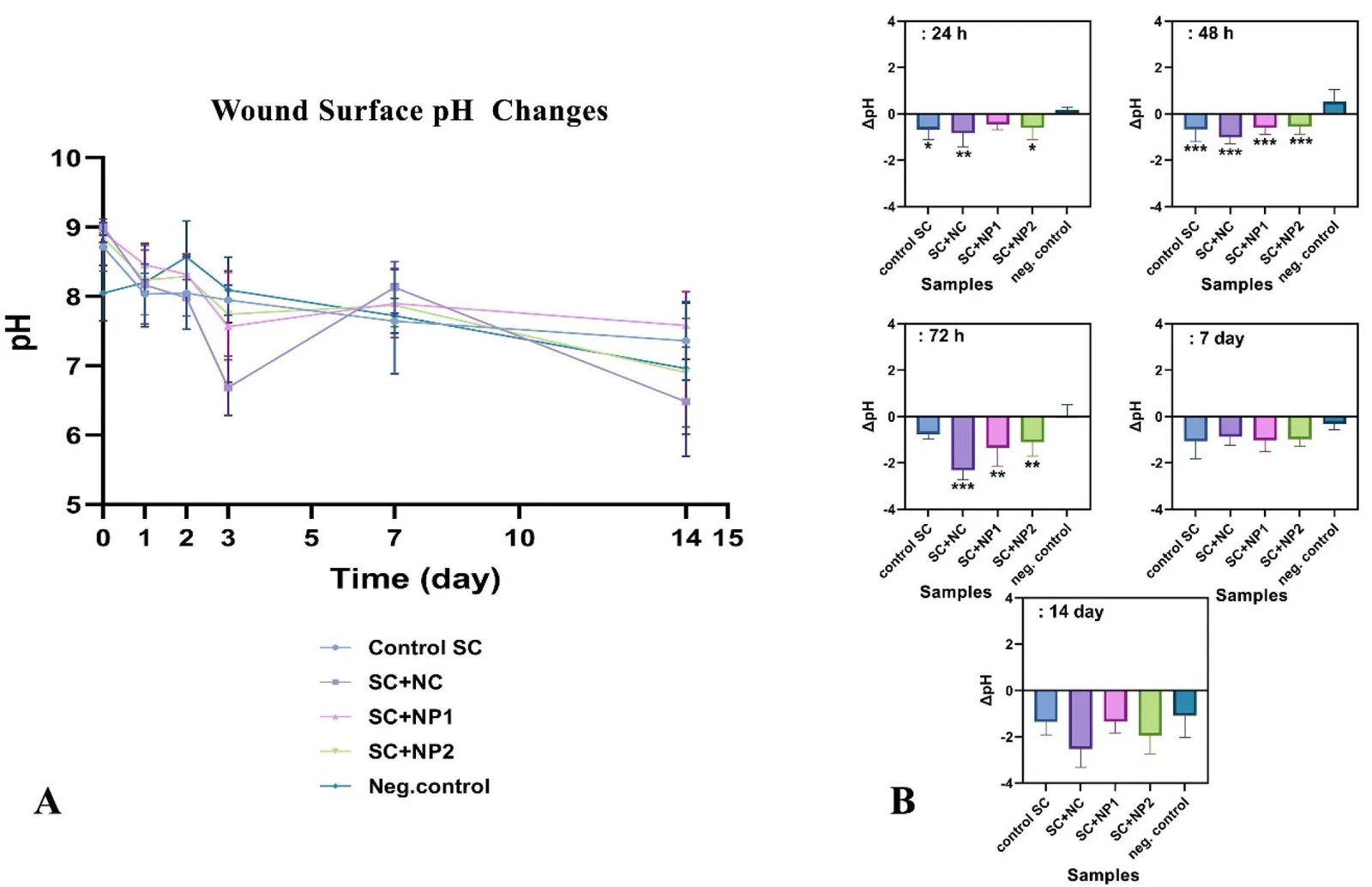
A) Changes in wound surface pH following the application of fish skin-derived acellular dermal matrix (FS-ADM) over a 14-day period. The graph depicts temporal pH variations among different experimental groups. pH measurements were recorded at baseline (day 0), 24, 48, and 72 hours, and on days 7 and 14 post-grafting. B) Bar graphs showing the magnitude of pH changes (ΔpH) in each group at 24 h, 48 h, 72 h, 7 days, and 14 days, relative to their respective baseline values. Data are presented as mean ± SD (n = 3). Statistical analyses were performed using one-way ANOVA followed by Tukey’s multiple comparison test to compare treatment groups and time points.; *P < 0.05, **P < 0.01, ***P < 0.001 compared to the control SC group

 Due to differences in the initial pH values at time zero, the initial pH was subtracted from the pH measured at each subsequent time point. The resulting ΔpH values were then compared between groups (Figure 4B).

 The delta pH value for the control SC group showed modest fluctuations, ranging from -0.684 at 24 hours to -1.365 at 14 days, indicating a relatively stable pH environment. In contrast, the SC + NC group exhibited a more pronounced decline, with delta pH dropping from -0.853 at 24 hours to -2.119 by day 14, reflecting a significant reduction. The SC + NP1 group showed a moderated decrease, while the SC + NP2 group showed notable decline over the same period.

 On day 7, the SC + NC group recorded the most substantial delta pH of -0.874. The SC + NP1 group showed slightly greater drop, and SC + NP2 group showed less delta pH decline. Overall, the data reveal distinct pH response patterns across the groups, emphasizing the therapeutic promise of the tested formulations.

####  Macroscopic Assessment

 As seen in Figure 5, the influence of FS-ADM combined with TA-loaded HA-nanogels on wound healing in rats was assessed. The experimental design included five groups, control SC group, SC + NC, SC + NP1, and SC + NP2 and negative control monitored at 0, 24, 48, 72 hours, and 7, 14 and 21 days to determine the therapeutic potential of the composite material.

**Figure 5:**
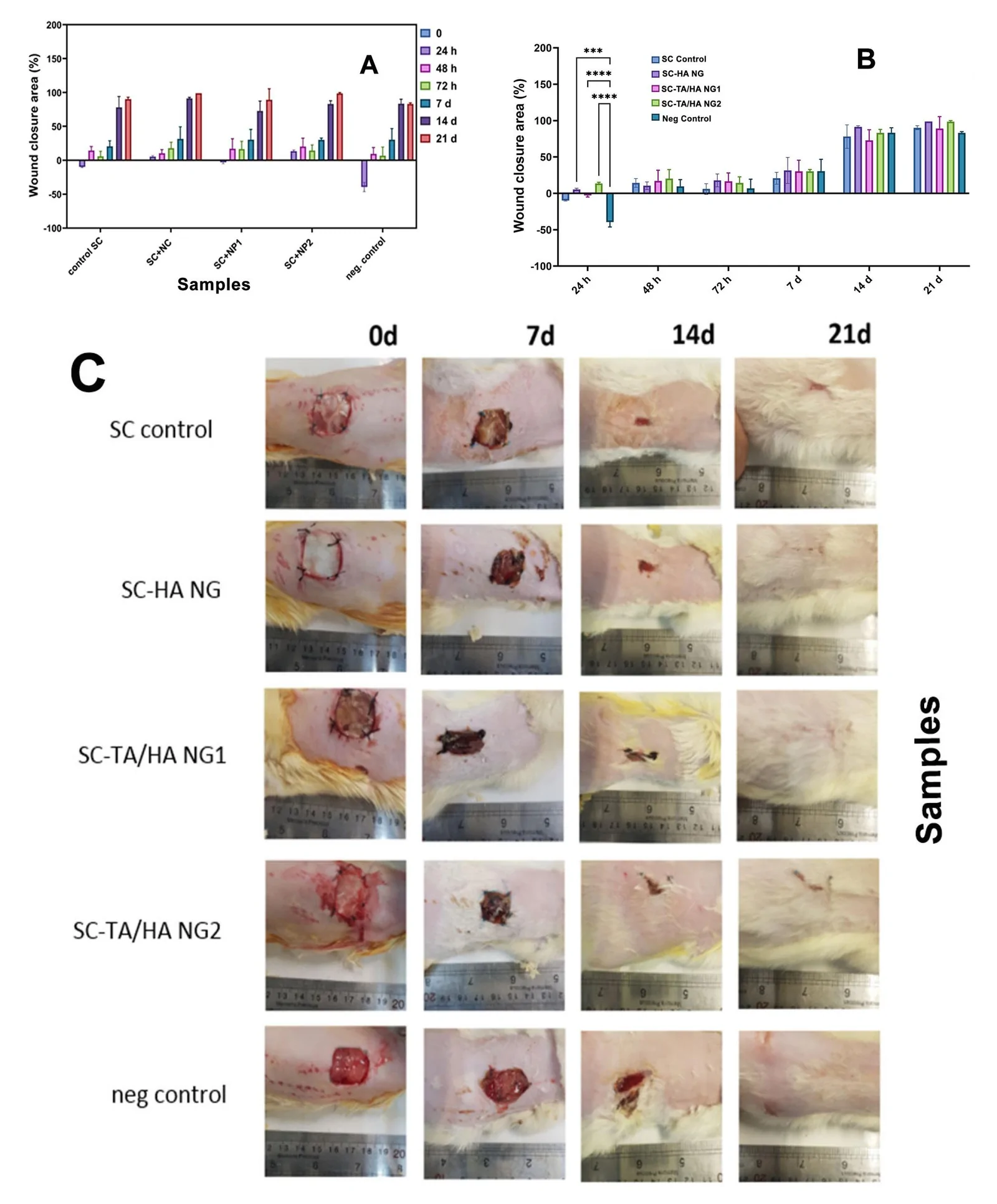
A) Statistical analysis of wound closure area in different groups (SC, SC + NC, SC + NP1, SC + NP2 and negative control group) at multiple time points (0, 24, 48, and 72 hours, and days 7, 14, and 21) following treatment B) Quantitative analysis of the percentage of wound closure in different groups at time points. Data are shown as mean ± standard deviation (SD). Statistical analysis was performed with one-way ANOVA and Tukey’s post hoc test for multiple comparisons. Significant differences were observed between groups at 24 hours (***P < 0.001, ****P < 0.0001). No significant differences were observed at other times. C) Macroscopic evaluation of wound healing progression in five groups on days 0, 7, 14, and 21 post-treatment. Visual observations support the quantitative data. Data are presented as mean ± SD (n = 3)

 At the 24-hour mark, the SC control group exhibited a wound expansion of 9.86%, SC + NC group showed modest healing with a 5.38% closure rate, SC + NP1 group demonstrated a slight regression, with a 3.00% closure rate, and SC + NP2 group achieved the most notable early improvement, closing 13.64% of the wound. These results indicate the SC + NP2 had the strongest initial effect, After 72 hours, SC improved slightly to 5.95%, suggesting slow recovery, the SC + NC group increased to 17.82%, likely due to sustained HA impact, indicating effective tissue repair, SC + NP1 reached 16.56%, showing notable progress, and SC + NP2 group reached 14.34%, possibly due to slower release kinetics. By day 21, SC achieved 90.18% closure, reflecting substantial healing over time, SC + NC reached 98.89%, confirming its long term efficacy, SC + NP! Reached 89.14%, trailing control group, and SC + NP2 closely matched SC + NC with 98.53% closure, indicating strong sustained performance. These findings highlight the superior long-term healing potential of nanogel formulations, suggesting future research should focus on optimizing their early and sustained therapeutic effects. The 21-day period was identified as optimal for maximum wound closure, with SC + NC and SC + NP2 delivering the best outcomes.

 The comparison of wound closure percentages between the groups shows a significant difference during the first 24 hours (***P < 0.001, ****P < 0.0001); however, in the subsequent stages of wound healing, no significant difference in wound closure was observed among the groups (Figure 5B).

####  Histopathologic Findings

 Histopathological analysis of the samples was conducted at 4 phases, day 3 (early inflammatory phase), day 7 (proliferative phase), day 14 (advanced healing phase), and day 21 (maturation phase).

Figure 6 shows the result of day 3, early inflammatory phase. All groups showed varying inflammation. Control SC showed marked edema, neutrophil infiltration, and fibrin accumulation with limited neovascularization. SC + NC showed reduced fibrin, enhanced angiogenesis, indicating faster inflammatory resolution, SC + NP1 group showed similar vascular and fibroblast activity to SC + NC group. SC + NP2 showed hemostasis with heavy fibrin and early neutrophil presence while negative control showed edema, bleeding, fibrin, and minimal immune cell infiltration.

**Figure 6:**
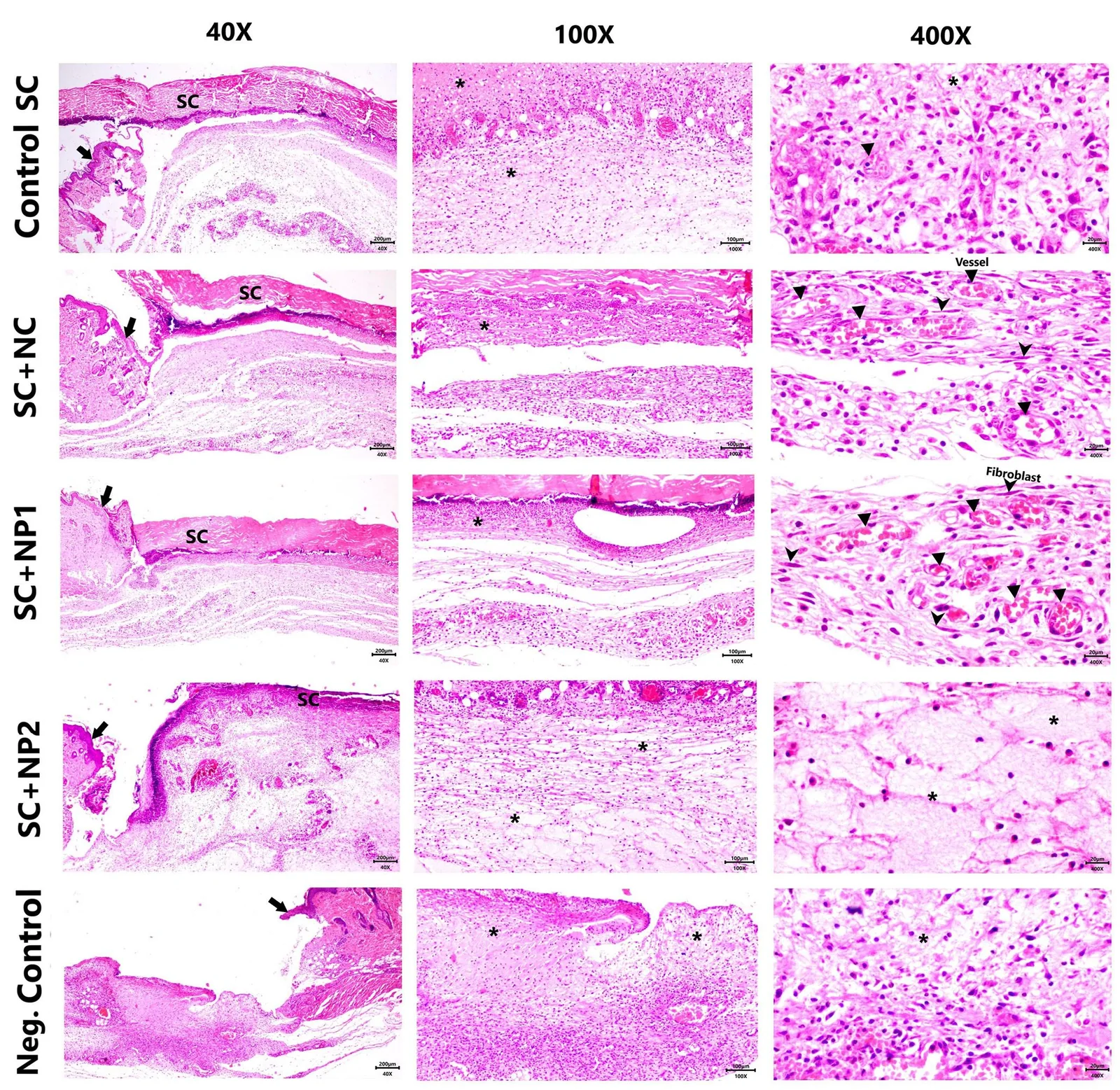
Histological analysis of wound tissues at day 3 post-injury. H&E-stained tissue sections from wound sites at 40X, 100X, and 400X magnifications. Control SC group shows scaffold placement with underlying fibrin accumulation and inflammatory infiltrate. SC + NC group demonstrates advanced inflammatory phase with reduced fibrin, increased neutrophils, and notable neovascularization. SC + NP1 group exhibits patterns similar to SC + NC. SC + NP2 group shows predominant hemostasis phase with marked fibrin presence. Negative Control group displays edema, hemorrhage, and sparse inflammatory cell infiltration. Asterisks (*): edematous and fibrinous regions; triangular arrows: blood vessels; arrowheads: fibroblasts; SC: scaffold; black arrows: intact epithelium at wound margin

 On day 7 (proliferative phase) granulation tissue formed in all groups (Figure 7). SC + NC: showed dense, organized tissue with active fibroblasts, fine collagen, and thick epithelium. SC + NP1 & SC + NP2 groups showed moderate granulation and neovascularization, epithelial coverage incomplete. SC (control) showed thin granulation with persistent inflammation. Negative control showed poor tissue organization and severe inflammation.

**Figure 7:**
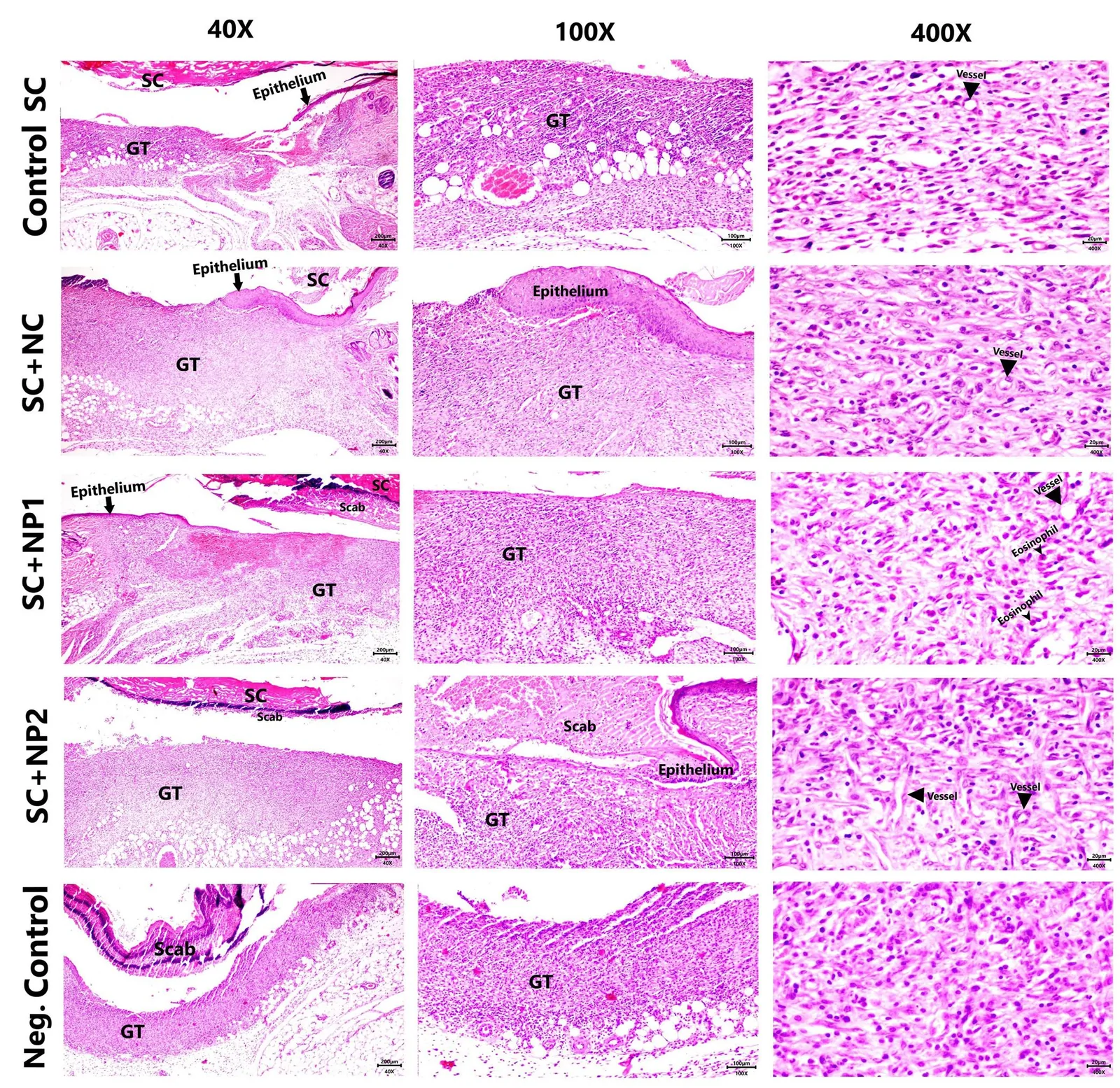
Photomicrographs of wound area at day 7. H&E-stained tissue sections, control SC group displays scaffold (SC) and regenerating epithelium at wound surface with underlying granulation tissue (GT) containing neovascularization. SC + NC group demonstrates enhanced healing with dense granulation tissue, organized fibroblasts, collagen deposition, and advanced epithelialization. SC + NP1 and SC + NP2 groups show surface scab formation with moderately dense granulation tissue and abundant vasculature, with SC + NP1 notably exhibiting distinct eosinophil infiltration (black arrowheads). Negative Control group shows a thin, irregular, immature granulation tissue and thick surface scab. Triangular arrows indicate blood vessels

 On day 14 (advanced healing phase, healing scores revealed significant differences (Figure 8). As can be seen SC + NC group showed complete epithelialization, keratinization, and mature scar tissue (score 7.67 ± 0.58). SC + NP2 group showed intermediate healing with partial epithelial coverage (score 6.00 ± 1.00). SC + NP1 group showed less efficient than SC + NC but better than control (score 5.00 ± 1.00). SC control group showed limited healing (score 3.33 ± 0.58). Negative control showed disorganized collagen, high fibroblast density, and persistent inflammation.

**Figure 8:**
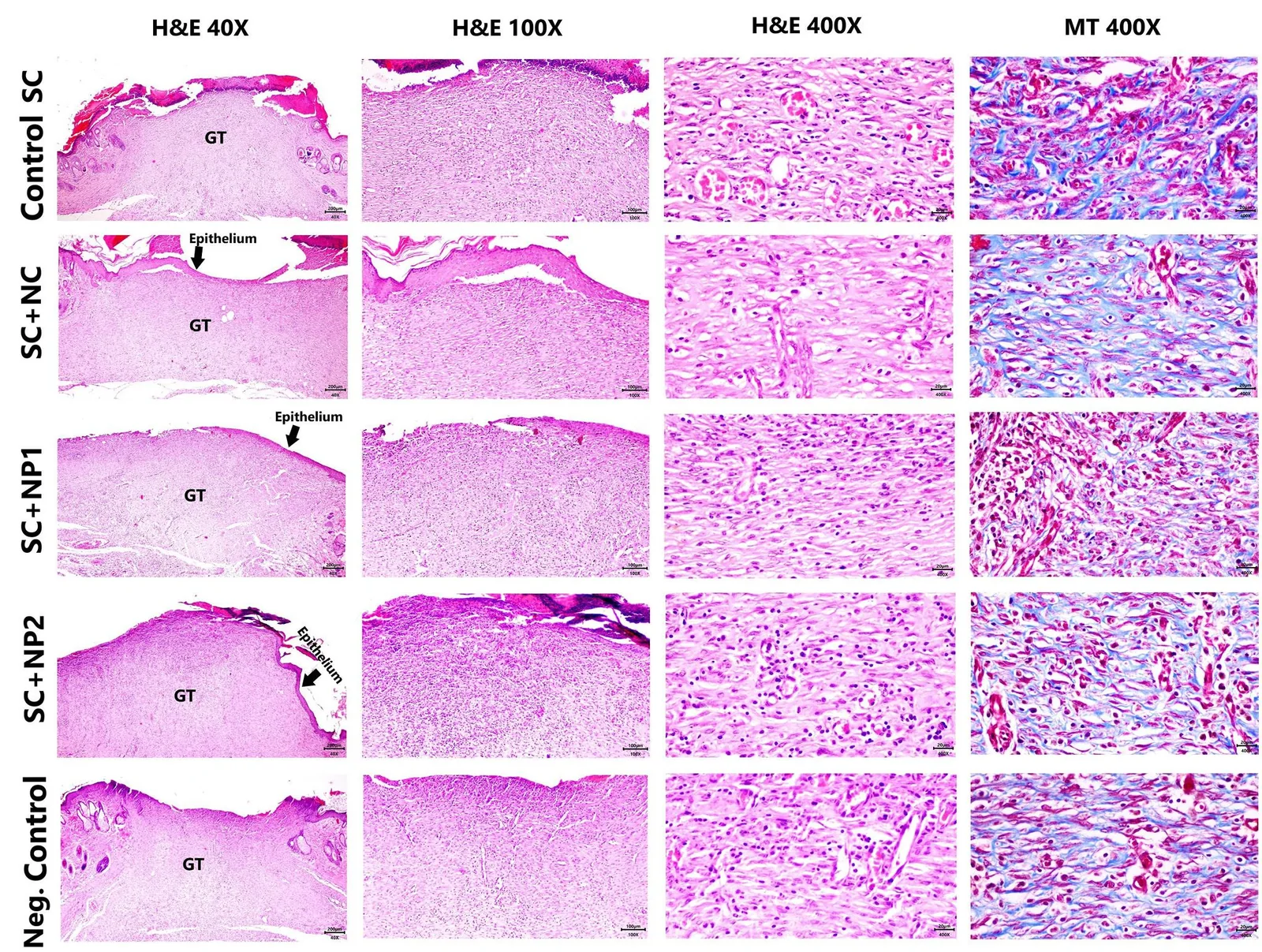
Histopathological comparison of wound healing across treatment groups at day 14 post-injury. Microscopic images with H&E staining at 40X, 100X, and 400X magnifications and Masson’s trichrome (MT) staining at 400X. Control SC group demonstrates hypertrophic granulation tissue (GT) with high-density irregular fibroblasts and abundant neovascularization with disorganized collagen fibers. SC + NC group exhibits optimal healing with thick keratinized epithelium formation and mature fibrous scar tissue featuring organized collagen alignment (blue in MT). SC + NP1 group shows thin non-keratinized epithelium with moderately mature granulation tissue, intermediate fibroblast density, and partially organized collagen deposition. SC + NP2 group presents a pattern similar to SC + NP1 but with enhanced granulation tissue maturity and focal epithelial keratinization. Negative Control group displays immature granulation tissue characterized by abundant fibroblasts, numerous blood vessels, and irregular collagen arrangement, indicating delayed healing progression. Scale bars: 100 μm (40X), 50 μm (100X), and 20 μm (400 X)

 On day 21 (maturation phase) final tissue regeneration and scar formation was assessed (Figure 9). As can be seen, SC + NC & SC + NP2 groups showed best outcomes with full epithelial regeneration, keratinization, and skin appendage restoration (scores: 9.67 ± 0.58 and 9.00 ± 1.00, respectively). SC + NP1 group showed moderate healing with complete epithelium but less organized collagen (score 6.67 ± 0.58). SC control group showed lower healing score (5.67 ± 0.58), minimal scar maturation and finally negative control group showed thin epithelium, disorganized connective tissue, modest healing (6.00 ± 2.00).

**Figure 9:**
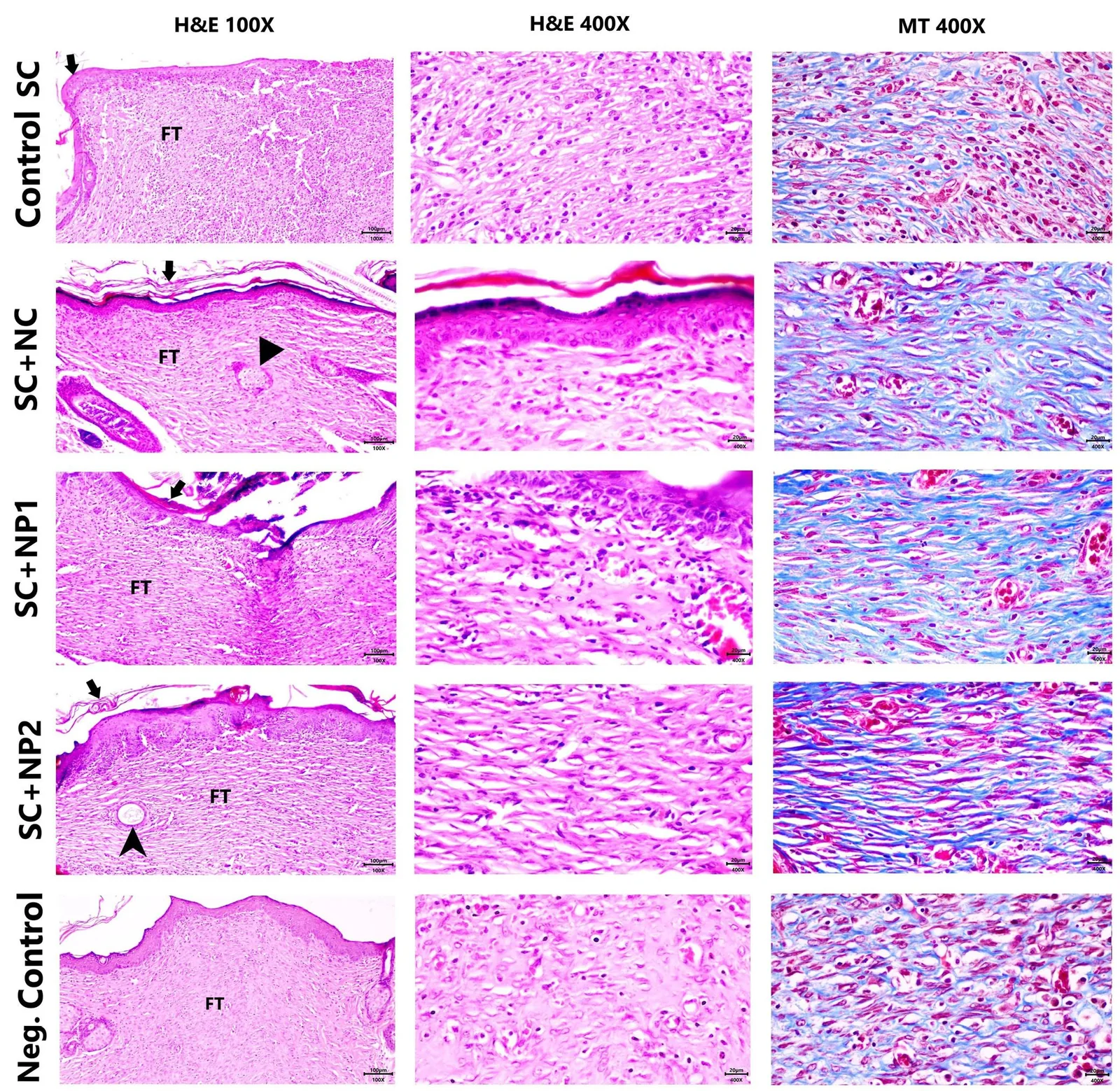
Histopathological evaluation of wound healing on day 21 post-injury. Representative microscopic images with H&E staining at 100X and 400X magnifications and Masson’s trichrome (MT) staining at 400X. Control SC group shows complete but minimally matured epithelium (black arrows) with underlying fibrous tissue (FT) containing parallel collagen fibers and moderate vascularity. SC + NC group exhibits optimal healing with thick keratinized epithelium, well-organized dense collagen bundles, and regenerated sebaceous glands (arrowhead). SC + NP1 group displays complete epithelialization with moderately organized collagen in the dermal layer. SC + NP2 group demonstrates advanced tissue remodeling with keratinized epithelium and regenerated hair follicles (arrowhead), similar to SC + NC group. Negative Control group shows complete but thin epithelium with disorganized collagen and relatively immature fibrous tissue. Scale bars: 50 μm (100X) and 20 μm (400X)

####  Quantitative Evaluation of Wound Healing

 Day 14 Analysis: Quantitative assessment of total histopathological healing scores at day 14 post-injury revealed significant differences among treatment groups. The control SC group exhibited the lowest healing score between treatment groups, whereas SC + NC achieved the highest, showing highly significant improvement over the negative control (*P* < 0.0001) and control SC (*P* < 0.001). The SC + NP1 group demonstrated a moderate but significant reduction compared to SC + NC (*P* < 0.05), yet remained superior to control SC. The SC + NP2 group showed intermediate healing; although differences with SC + NC and SC + NP1 were not significant, its performance exceeded that of control SC (*P* < 0.05).

 Overall, by day 14, all treated groups outperformed the negative and control SC groups, with SC + NC exhibiting the most pronounced wound healing efficacy (Figure 10).

**Figure 10:**
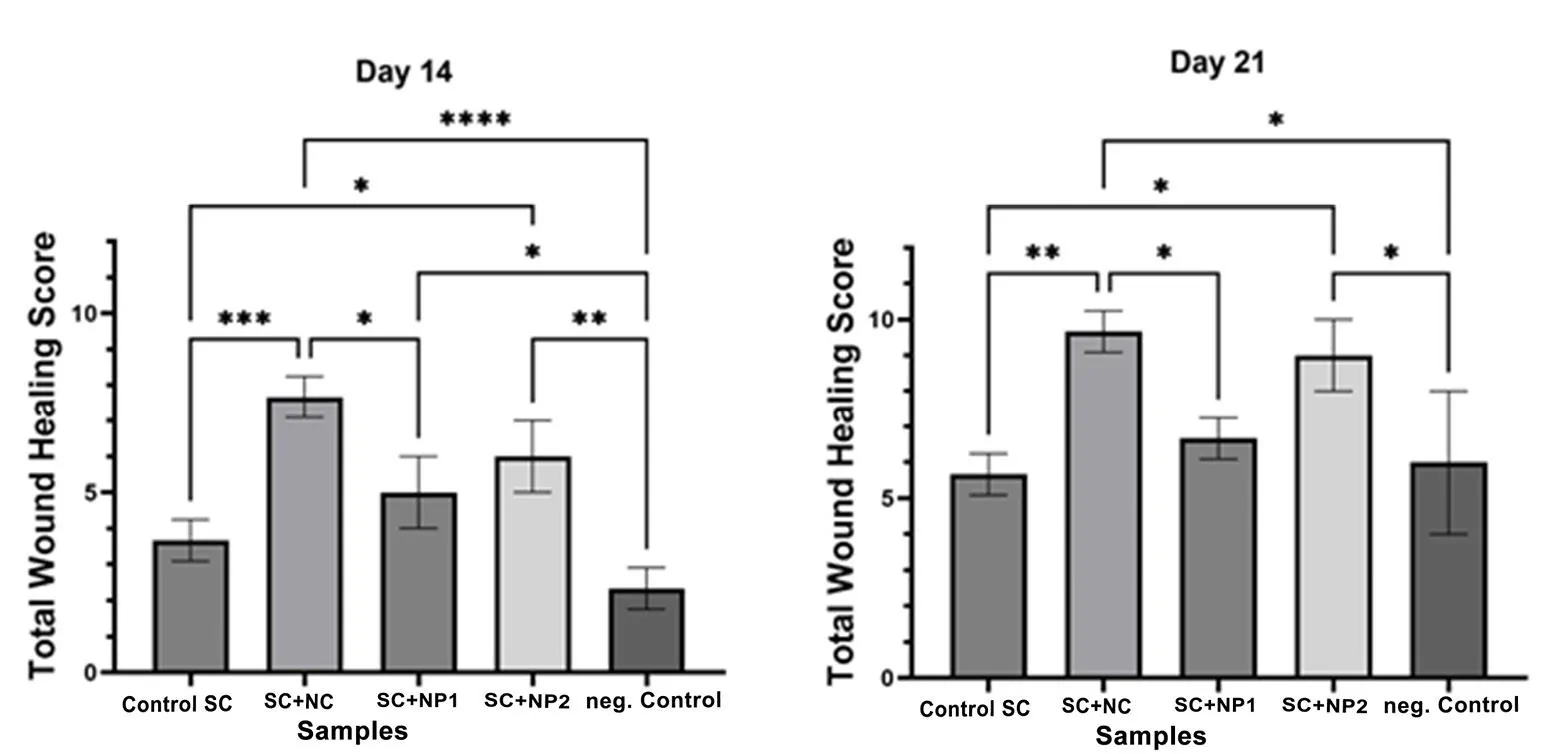
Comparative analysis of total wound healing scores at days 14 and 21 post-injury across treatment groups. Bar graphs show quantitative evaluation of wound healing based on a comprehensive scoring system incorporating epithelialization, granulation tissue maturation, collagen organization, and skin appendage regeneration. At day 14, SC+NC group demonstrated significantly higher healing scores compared to control SC, SC + NP1, and negative Control groups (*P < 0.05, **P < 0.01, ***P < 0.001, ****P < 0.0001). By day 21, both SC + NC and SC + NP2 groups achieved the highest healing scores, significantly outperforming other groups (*P < 0.05, **P < 0.01). Data are presented as mean ± SD (n = 5). Statistical analyses were performed using one-way ANOVA followed by Tukey’s multiple comparison test

 Day 21 Analysis: At day 21, total histopathological scores again showed significant variation across treatments. The control SC group retained the lowest healing capacity, while SC + NC achieved the highest score, differing significantly from the negative control (*P* < 0.05) and SC control (*P* < 0.01). The SC + NP1 group exhibited a significant reduction relative to SC + NC (*P* < 0.05). The SC + NP2 group demonstrated substantial healing comparable to SC + NC, with no significant difference between them.

 Collectively, these results indicate that by day 21, SC + NC and SC + NP2 achieved the most effective wound healing, while SC + NP1 showed relatively lower efficacy. Healing progression was enhanced in all treatment groups compared to day 14 (Figure 10).

## Discussion

 The application of carp fish skin integrated with HA nanogels containing various acidifying agents demonstrated a sustained and effective reduction in pH levels, both in vitro and in vivo, despite the buffering capacity of physiological environments. In vitro release study demonstrated a consistent pH drop of approximately 1.5 units over a 7-day period. Remarkably, this acidification effect was maintained in vivo, even in the presence of wound exudate, which typically neutralizes acidic agents. Among the treatment groups, SC + NP1 group showed the most significant pH reduction by day 7, while SC + NC group exhibited the highest cumulative pH change by day 14. This suggests that the SC + NC formulation may release its acidifying components more gradually, leading to delayed but sustained acidification. Interestingly, the addition of TA to the HA nanogel did not enhance pH reduction beyond that achieved by HA nanogel alone. This limitation is likely due to ionic interactions between TA and the HA matrix, which may restrict the release of free acidic components.

 Histopathological evaluation revealed distinct healing patterns among the treatment groups. By day 21, both SC + NC and SC + NP2 groups demonstrated superior tissue regeneration. These groups achieved complete epithelial restoration with proper keratinization, mature scar formation characterized by well-organized collagen fibers, and regeneration of skin appendages, sebaceous glands in SC + NC and hair follicles in SC + NP2. These advanced healing outcomes correlated with their enhanced pH modulation profiles, underscoring the therapeutic value of controlled acidification in wound healing.

 The SC + NP1 group exhibited intermediate healing results. Although epithelialization was complete, collagen deposition remained moderately organized, and vascular remodeling was limited. In contrast, the control SC group, despite achieving full epithelial coverage, showed poor scar maturation. Collagen fibers were aligned but sparsely packed, vascularity was moderate, and inflammatory cells persisted, leading to significantly lower healing scores. The negative control group, which lacked the bioactive scaffold and acidifying component tissue, showed wound contraction with thin epithelial layers and disorganized connective tissue, reflecting suboptimal healing.

 Alternative acidifying agents such as ascorbic acid or acetic acid may offer improved efficacy over TA. Several acids, including hyaluronic, hypochlorous, citric, and acetic acids, are already used topically in managing diabetic foot infections.^37^ Among these, HA stands out due to its dual functionality: it is highly water-absorbent and possesses antimicrobial properties, making it a particularly promising candidate for wound acidification and wound healing enhancement.^38^

 Several recent reviews and studies have explored hyaluronic acid (HA)–based biomaterials for wound healing, emphasizing hydrogels, nanofibers, and hybrid nanocomposites. Comprehensive reviews by Ahmed et al.,^39^ Nandhini et al.,^40^ and Kushwaha et al.^41^ summarized the state of HA-based and nanomaterial-assisted wound dressings. However, these works primarily focus on chemically crosslinked or metal-based systems and do not identify polyelectrolyte complex (PEC) nanoparticles as a distinct, self-assembled, and biocompatible platform. The PEC nanocarrier system developed in this study therefore represents a unique extension of HA-based wound therapeutics, eliminating the need for toxic crosslinkers while preserving the polymer’s native biocompatibility and biodegradability.

 Earlier work by Silva et al.^32^ reported HA–lysine PEC nanoparticles for dermal filling applications, focusing mainly on volumetric and mechanical properties. In contrast, our HA-based PEC nanoparticles were specifically engineered as functional nanocarriers to achieve gradual and controlled acid release capable of actively modulating the wound microenvironment. This approach enables pH-dependent biochemical regulation while maintaining structural compatibility with biological tissues.

 Within the context of pH-regulated wound healing, several studies have acknowledged the importance of local acidity in promoting tissue regeneration Sim et al.^42^ demonstrated the beneficial effects of acidic pH on wound closure but did not provide quantitative or temporal wound-surface pH measurements. Their related in vitro investigation^43^ utilized acidified buffers without nanoparticle incorporation or in vivo validation. Similarly, Abid et al.^44^ examined pH reduction in vitro to inhibit bacterial growth and biofilm formation, while Cui et al.^45^ introduced microfluidic microgel systems to control wound pH—an approach that, although innovative, requires specialized fabrication equipment and lacks translational practicality for large-scale use.

 Carp fish, as an endemic fish in Iran, is readily available, has a clean white appearance, and maintains a stable texture for suturing. Moreover, our treatment protocol effectively removed nuclear and other immunogenic components, and in vivo studies confirmed the absence of infection markers and endotoxins. Carp skin also demonstrated excellent moisture management by absorbing wound exudate efficiently.

 The FS-ADM integrated with HA nanogel was developed without chemical crosslinking, minimizing cytotoxicity and preserving biocompatibility. This natural scaffold is biodegradable and biocompatible and supports healing without provoking adverse skin reactions. Compared to open wounds, this dressing induces direct contact with external bandages, thereby lowering the risk of skin immunologic reaction and infection. Additionally, the hydrolyzed proteins in fish skin have been reported to possess antimicrobial properties, further contributing to infection control.^38^

 Bacterial colonization observed on day 3 across all groups was likely due to initial wound manipulation. However, subsequent observations showed that microbial contamination was eliminated in all treated groups, while the open wound group continued to exhibit bacterial presence even at days 14 and 21. This supports the hypothesis that the scaffold possesses antibacterial properties. The acellular fish skin appears to act as a physical barrier against microbial invasion, although further studies are needed to confirm this effect definitively. It is well established that wounds with elevated alkaline pH, whether acute of chronic, heal more slowly s than those with neutral or slightly acidic environments.^46^

 The excisional wound model was selected for its ease of suturing and clinical relevance. Future research should explore the application of medicated ADM in more complex wound types, such as diabetic or burn wounds, where pH dysregulation is more pronounced.

 Clinical observations, such as the study by Wang et al.^47^ on diabetic foot ulcers, monitored pH variation during healing using litmus-based colorimetric methods. Despite offering qualitative insights, this approach lacked precise quantification and did not include active pH modulation. In contrast, our study utilized a surface pH meter for accurate and repeatable measurement, enabling direct assessment of pH changes both in vitro and in vivo. The HA-based PEC nanocarrier facilitated sustained acid release, maintaining a mildly acidic wound environment that supports healing while preventing periwound dermatitis.

 Historically, diabetic foot ulcers have posed a significant challenge due to the limitations of conventional treatments like antibiotics and standard wound care. Infection remains the primary barrier to effective healing in these cases. Identifying a topical antiseptic that is safe, potent, and accelerates healing while controlling infection is a major clinical need.^48^ Most pathogenic bacteria thrive at neutral pH (~7.0), whereas acidic environments (pH < 6.0) inhibit their growth and proliferation. Thus, acidifying agents not only promote healing but also serve as antimicrobial barriers.

 Previous studies have highlighted the limitations of standard antiseptics due to their cytotoxicity. In contrast, acids such as citric acid, hyaluronic acid, hypochlorous acid, and acetic acid, ranked in descending order of efficacy, offer safer and more effective alternatives for managing diabetic foot infections.

 In addition to its local availability, carp skin offers several biological and translational advantages that make it a promising alternative to conventional acellular dermal matrix (ADM) sources such as porcine, tilapia, and cod. Carp skin possesses a dense, well-organized type I collagen network closely resembling the human dermal extracellular matrix, which supports excellent cellular adhesion and migration.^49^ Its bioactive lipids and omega-3 fatty acids contribute to anti-inflammatory activity and promote angiogenesis during wound repair.^50^ Compared with marine-derived ADMs such as cod or tilapia, carp represents a freshwater source with a distinct lipid and peptide composition, reducing allergenic potential and simplifying sustainable sourcing. Moreover, unlike porcine ADM, which may be limited by religious or cultural restrictions,^51^ carp-based matrices are broadly acceptable across different populations. Collectively, these features highlight carp skin as a robust, biocompatible, and ethically versatile ADM source with strong translational potential for wound-healing applications.

 Recent advances in smart wound dressings have introduced pH-responsive “smart bandages” that can sense and adapt to changes in the wound microenvironment. Similar to the concept presented by Pan et al. ^52^ these systems are designed to respond dynamically to local pH variations, releasing therapeutic agents or providing diagnostic feedback based on wound status. Such pH-responsive platforms represent an emerging direction in wound-care research, aiming to create self-regulating or feedback-driven healing environments. In line with this approach, our PEC nanocarrier system provides intrinsic pH sensitivity and gradual acid release without requiring complex sensors or external stimuli, offering a simpler and fully biocompatible alternative for future intelligent wound-dressing applications.

 Altogether, the integration of the HA-based PEC nanocarrier with the fish skin–derived acellular dermal matrix (FS-ADM) in this study exemplifies a multi-modal wound-healing strategy. The combined system leverages chemical modulation of the wound microenvironment via controlled acidification and the physical support provided by the natural extracellular matrix scaffold. This synergistic design offers a promising approach for the treatment of deep or chronic wounds, combining biochemical, structural, and physicochemical mechanisms to enhance tissue regeneration, reduce periwound complications, and accelerate healing.

 Based on these findings, our integrated approach—combining carp fish skin ADM with pH-modulating nanogels—represents a promising therapeutic strategy for chronic wounds, particularly those with alkaline profiles like diabetic ulcers. Future investigations should focus on validating this approach in diabetic and burn wound models, where pH imbalance plays a critical role in delayed healing.

## Conclusion

 This study provides evidence that acellular dermal matrices derived from carp fish skin- (FS-ADM), especially when integrated with hyaluronic acid (HA)–based nanogels, offer a promising strategy for promoting wound repair. The developed nanogels showed sustained pH-reducing effects, controlled degradation, and excellent biocompatibility. In vivo studies revealed that FS-ADM combined with HA nanogel (SC + NC) markedly enhanced epithelial regeneration, collagen alignment, and restoration of skin appendage. Interestingly, HA nanogels lacking Tannic acid (TA) performed better than those containing TA, indicating that the scaffold itself plays a crucial role in shaping the wound microenvironment. These results highlight the therapeutic potential of FS-ADM paired with pH-responsive nanogels for treating chronic wounds, such as diabetic ulcers. Further research is needed to confirm these findings in clinically relevant settings and assess their applicability in medical practice.

## Availability of Data and Materials

 All data analyzed during this study are included in this published article

## Competing Interests

 The authors declare that they have no competing interests

## Ethical Approval

 Animal studies were fulfilled according to the codes of ethics declared by the Iran National Committee for Ethics in Biomedical Research (IR.TUMS.TIPS.REC.1398.210).

## Supplementary File

Supplementary file contains Figure S1 and Figure S2.
