# Supplementary material for: Fish Skin-Derived Acellular Matrix with pH-Modulating Nanogels for Wound Healing
Source: Adv Pharm Bull. 2026 May 10;16(2):387–402. doi: 10.34172/apb.46166 (PMC13745438; doi:10.34172/apb.46166)
Supplement: Supplementary file 1 [file apb-16-387-s001.pdf]

Adv Pharm Bull 2026;16(2):387-S1
doi:10.34172/apb.46166
https://apb.tbzmed.ac.ir

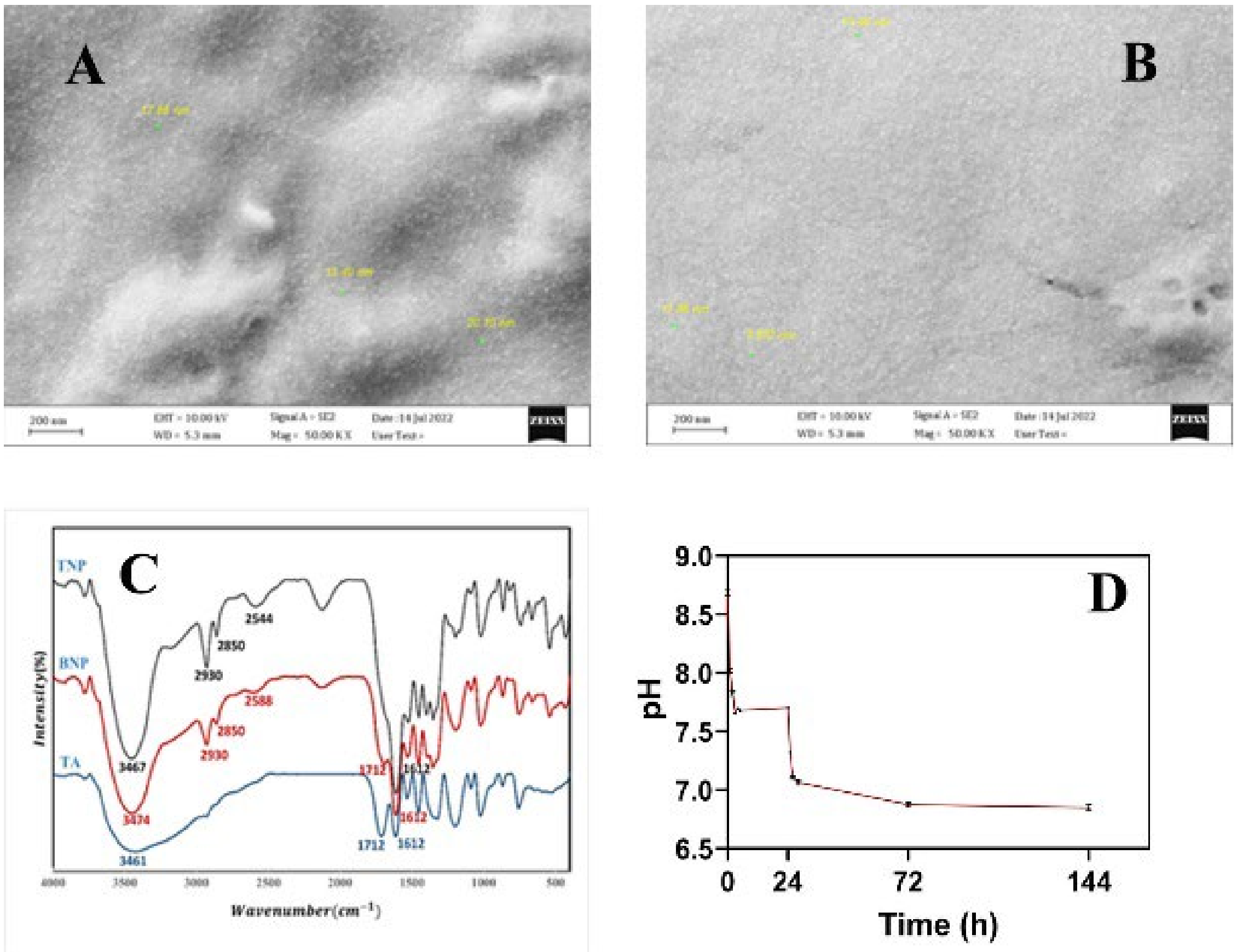

Figure S1. A,B) FSEM images of nanogels the scale bar represents 200nm, C) FTIR graphs of tannic acid powder (TA), HA nanogel as blank (BNP), TA-loaded HA nanogel (TNP), D) pH changes due to HA, TA Release, and data are presented as mean ± SD (n = 3).

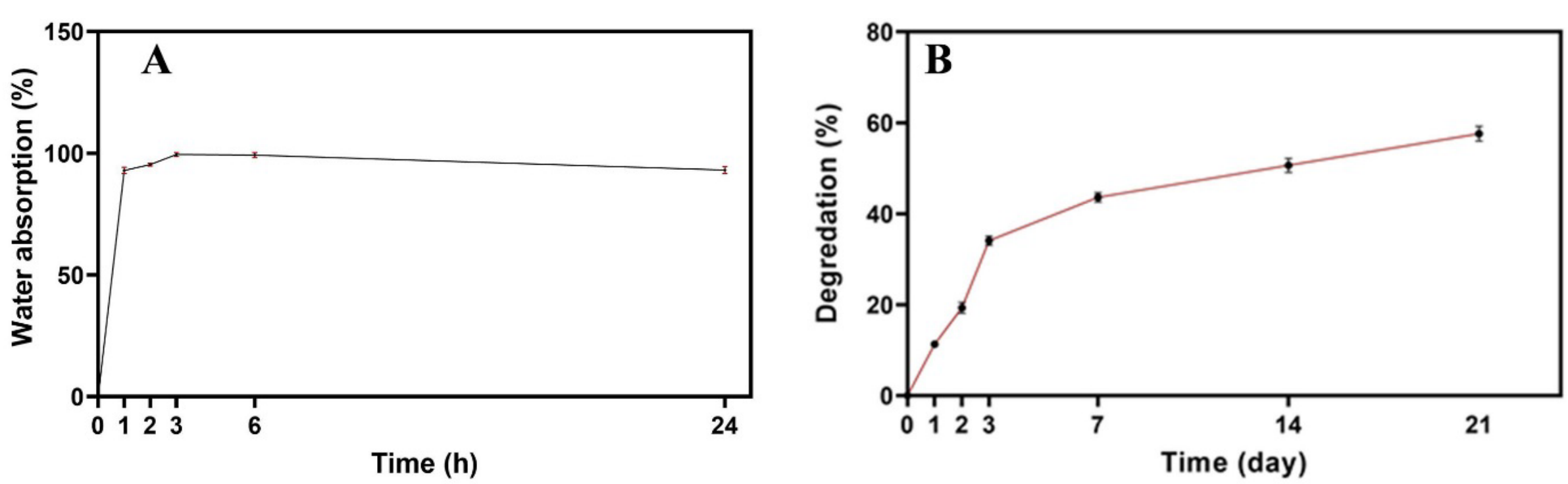

Figure S2. A) Diagram of water absorption capacity in Carp fish samples of FADMs. B) Diagram of degradation percent in Carp fish samples of FADMs, Data are expressed as mean ± SD (n = 3).
